# Poly(ADP-ribose) polymerase-1 and its ambiguous role in cellular life and death

**DOI:** 10.15698/cst2023.01.275

**Published:** 2023-01-23

**Authors:** Maria Castedo, Antoine Lafarge, Guido Kroemer

**Affiliations:** 1 Equipe 11 labellisée par la Ligue contre le Cancer, Université de Paris Cité, Sorbonne Université, INSERM U1138, Centre de Recherche des Cordeliers, 75006 Paris, France.; 2 Metabolomics and Cell Biology Platforms, Gustave Roussy Cancer Campus, Villejuif, France.; 3 Faculté de médecine, Université de Paris Saclay, Kremlin Bicêtre, France.; 4 Institut du Cancer Paris CARPEM, Department of Biology, Hôpital Européen Georges Pompidou, Assistance Publique-Hôpitaux de Paris, Paris, France.

**Keywords:** PARP1 inhibition, DNA damage, parthanatos, cancer, neurological diseases, aging, cellular stress response

## Abstract

The deletion of the gene coding for poly(ADP-ribose) polymerase-1 (PARP1) or its pharmacological inhibition protects mice against cerebral ischemia and Parkinson's disease. In sharp contrast, PARP1 inhibitors are in clinical use for the eradication of vulnerable cancer cells. It appears that excessive PARP1 activation is involved in a specific cell death pathway called parthanatos, while inhibition of PARP1 in cancer cells amplifies DNA damage to a lethal level. Hence, PARP1 plays a context-dependent role in cell fate decisions. In addition, it appears that PARP1 plays an ambiguous role in organismal aging.

In biology, the integration of intracellular circuitries is achieved by the multifunctionality of molecules, molecular complexes and organelles [1]. Poly(ADP-ribose) polymerase-1 (PARP1) exemplifies a multitasking protein that fulfills several signaling functions in the context of cellular stress response, senescence and aging, as well as pathological cell death. Here, we will briefly summarize the multiple functions of PARP1 **(Figure 1)**.

**Figure 1:**
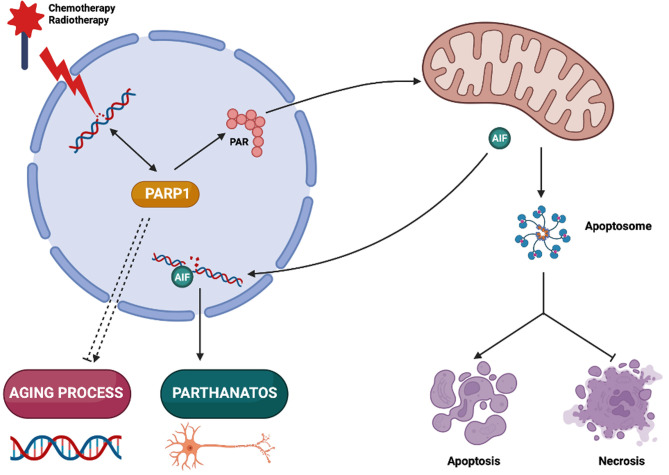
FIGURE 1. PARP1 context-dependent functions in cellular life and death. Poly(ADP-ribose) polymerase-1 (PARP1) is a DNA repair enzyme activated in cancer cells treated with DNA damaging agents, and pharmacological PARP1 inhibitors can be used to improve chemotherapy and radiotherapy efficacy. PARP1 inhibition may also have neuroprotective effects by decreasing parthanatos-induced neuronal death. However, the ambivalent role of PARP1 in organismal aging and cell death modalities should be considered in view of the possible side effects of long-term treatments with PARP1 inhibitors. PARP1, Poly(ADP-ribose) polymerase-1; PAR: Poly(ADP-ribose); AIF: apoptosis inducing factor.

## PARP1 IN DNA REPAIR: A TARGET FOR ANTICANCER DRUGS

PARP1 is best known as is a DNA repair enzyme present in the nuclei of mammalian cells. In response to single-strand DNA breaks, PARP1 initiates the synthesis of poly(ADP-ribose) (PAR) chains from nicotinamide adenine dinucleotide (NAD^+^). This process results in the covalent attachment of PAR to multiple proteins. PAR then acts as a signal for the recruitment and the activation of other DNA repair enzymes [2]. Logically, cancer cells that are treated with DNA damaging agents (such as radiotherapy or chemotherapeutic cytotoxicants such as cisplatin) must activate PARP1 to survive. Indeed, cisplatin-resistant cells upregulate PARP1 activity and hence contain higher PAR levels in their nuclei [3]. This is clinically relevant because high PAR levels detected by immunohistochemistry have a poor prognostic impact in several cancer types, correlating with reduced immunosurveillance [3–6]. In addition, BRCA2- and other homologous recombination-defective cells have increased PARP1 activation meaning that cell survival relies on a constitutive activation of PARP1 [7].

Pharmacological PARP1 inhibitors can be used to sensitize cancer cells to chemotherapy and radiotherapy [8–10]. Moreover, cells relying on constitutive PARP1 activation become sensitive to monotherapy with PARP1 inhibitors [3, 6, 7]. Indeed, several PARP1 inhibitors such as niraparib, olaparib, rucaparib and talazoparib have been clinically approved for the treatment of oncological patients with germline mutations in BRCA1/2, as well as for epithelial ovarian, fallopian tube and primary peritoneal cancer [9,10]. These small-molecule inhibitors interact with the binding site of NAD^+^ and inhibit the synthesis of PAR chains. However, the cytotoxic effect of PARP1 inhibitors against cancer cells is mostly mediated by the trapping of PARP1 at sites of DNA damage that generates stalled replication forks during the S phase of the cell cycle [11–13]. Therefore, novel PARP1 inhibitors with increased trapping capacity are promising candidates to target cancer cell death [14]. Of note, inhibition of PARP1 does not only have cancer cell-autonomous effects but also stimulates T lymphocyte-mediated anticancer immune response through yet-to-be-elucidated mechanisms [6, 12, 15]. Thus, combining PARP1 inhibition with immune checkpoint blockade holds promise for the treatment of ovarian cancer patients [16].

## PRO-DEATH ACTIVITY OF PARP1 IN PARTHANATOS

Ted and Valina Dawson described a cell death modality relying on the overactivation of PARP1 and PAR-induced mitochondrial outer membrane permeabilization (MOMP) that they coined “parthanatos” [17]. In this lethal pathway, oxidative or nitrosative stress results in the activation of PARP1. PAR accumulating at mitochondrial membranes then triggers the release of the intermembrane protein apoptosis inducing factor (AIF, official gene/protein name AIFM1) that subsequently interacts with parthanatos-associated apoptosis-inducing factor (AIF) nuclease (PAAN), also known as macrophage migration inhibitor factor (MIF). PAAN is a member of the PD-D/E(X)K nuclease family and acts as a final executioner in parthanatos. The genetic depletion or pharmacological inhibition of PARP1 and PAAN prevents neurodegeneration of dopaminergic neurons induced by stereotactic intrastriatal injection of α-synuclein preformed fibrils, systemic administration of the Parkinsonian neurotoxin 1-methyl-4-phenyl-1,2,3,6-tetrahydropyridine (MPTP) or overexpression of the parkin substrate aminoacyl-tRNA synthetase complex interacting multifunctional protein-2 (AIMP2) [18–20]. Parthanatos has been involved in rodent models of ischemic stroke in which PARP1 inhibitors given within 4 to 6 hours after middle cerebral artery occlusion (MCAO) confer neuroprotection [21]. Other disease models in which PARP1 inhibitors have cytoprotective effects include Alzheimer's disease (AD), Huntington's disease (HD) and amyotrophic lateral sclerosis (ALS) [22], experimental retinal detachment [23], as well as ischemia/reperfusion injury of the kidney or the liver [24, 25]. Parthanatos has also been involved in human psoriasis in which PARP1 is overactivated downstream of the NAD^+^ generating enzyme nicotinamide phosphoribosyltransferase (NAMPT) and the translocation of AIF from mitochondria to nuclei can be observed in skin lesions [26]. As an aside, cancer cells can die in response to specific drugs or drug combinations in a PARP1-dependent fashion [27–29]. Altogether, it appears that, outside of the realm of oncology, PARP1 inhibition might be useful for the treatment of diseases, many of which are caused by the acute, massive or chronic, insidious loss of neurons.

## PRO-APOPTOTIC CLEAVAGE AND INACTIVATION OF PARP1

Apoptosis usually involves two major hierarchically related events, namely (i) mitochondrial membrane permeabilization (MMP) and (ii) activation of specific set of proteases, which are called caspases. MMP may occur to variable proportions as mitochondrial inner membrane permeabilization (MIMP) and MOMP, leading to the arrest of oxidative phosphorylation and other essential metabolic functions, hence usually sealing the cell's fate [30, 31]. MOMP causes the release of proteins that are found in the intermembrane space or are loosely attached to the outer surface of the inner membrane into the cytosol. One prominent example is cytochrome *c* which, once released from mitochondria, interacts with APAF1 to stimulate the formation of the apoptosome, which is a caspase-9 activation complex [32]. Caspase-9 is an initiator caspase that then activates other caspases such as caspase-3 and caspase-7 that are effector caspases and destroy multiple intracellular proteins, hence dismantling the cell from inside [31]. When caspases are inhibited, MMP causes cell death, though without the morphological appearance of apoptosis with nuclear pyknosis, rounding of the cellular contours, shrinkage of the cytoplasm and formation of apoptotic blebs. Rather, MMP without subsequent caspase activation results in necrotic cell death with cellular oncosis, organellar swelling and early plasma membrane rupture [33]. In this context, it is important to note that PARP1 is a prominent caspase-3/7 substrate and that caspase-digested PARP1 loses its enzymatic function. Thus, when cells die in the absence of caspase activation, PARP1 tends to become activated by damaged DNA, hence futilely consuming ATP and NAD^+^, which contributes to the lethal bioenergetic crisis that culminates in necrosis. In this context, the genetic or pharmacologic inhibition of PARP1 favors the apoptotic rather than necrotic demise of dying cells [34, 35]. Thus, in certain situations, the activity of PARP1 does not determine the death/life decision itself but rather affects the propensity to succumb to one or the other lethal subroutine: apoptosis or necrosis.

## PRO-AGING AND ANTI-AGING EFFECTS OF PARP1

PARP1 is necessary for genomic stability, suggesting that its inhibition should derive genomic instability, which is one of the principal hallmarks of aging as well as of cancer. Indeed, transgenic PARP1 overexpression in basal skin keratinocytes from mice suppresses skin papilloma formation in a two-stage skin carcinogenesis protocol [36]. In Drosophila, conditional overexpression of PARP1 in the imago increases median lifespan of females and the maximum lifespan of males [37]. In mice, knockout of PARP1 accelerates aging and causes the precocious manifestation of spontaneous carcinogenesis, as well as a shift to a higher frequency of epithelial cancers of the lung, liver and uterus [38]. In patients with solid tumors, treatment with PARP1 inhibitors, results in an increased incidence of myelofibroblastic syndrome (which is a typical age-associated condition) and acute myeloid leukemia (which often develops from myelofibrosis) [39]. Possible mechanisms accounting for an age-related decline in PARP1 activity include a progressive depletion of the NAD^+^ [40]. This reduces PARP1 activity not only because NAD^+^ is an essential PARP1 substrate, but also due to the reduced binding of NAD^+^ to the protein deleted in breast cancer 1 (DBC1), which then engages in an inhibitory interaction with PARP1 [41]. Altogether, the aforementioned findings suggest that PARP1 has an antiaging function.

In sharp contrast, there are also arguments in favor of a pro-aging activity of PARP1. Thus, pharmacological PARP1 inhibition rescues the short lifespan of hyperglycemic *Caenorhabditis elegans* [42]*,* improves neurovascular and cognitive parameters in aging mice [43], and ameliorates cardiac performance in aging rats in which it also enhances acetylcholine-induced, nitric oxide-mediated vascular relaxation [44]. Conversely, mice expressing a human PARP1 transgene exhibit reduced healthspan and lifespan, accompanied by reduced hair growth and premature manifestation of inflammation and age-associated pathologies, such as anemia, adiposity, cardiomyopathy, dermatitis, hepatitis, kyphosis, nephropathy, pneumonitis, as well as an increase in the incidence of carcinomas [45]. The mechanisms accounting for these pro-aging effects of human PARP1 in mice are elusive. However, they plead for an ambiguous, dose-dependent implication of PARP1 in the aging process.

## CONCLUSION

Pharmacological inhibition of PARP1 may kill those cancer cells that are undergoing constant endogenous DNA damage due to defects in DNA repair enzymes as well as cells that are being exposed to DNA damaging irradiation or cytotoxicants. Paradoxically, PARP1 inhibition has potent cell death-inhibitory effects in other contexts, in particular against parthanatos, a non-apoptotic modality of cellular demise that relies on the enzymatic overactivation of PARP1 and that appears to be particularly prevalent in neurological diseases. In addition, PARP1 inhibition may accelerate or retard age-related pathologies, suggesting that PARP1 has an ambiguous role in aging as well. This consideration is important in view of the possible side effects of long-term treatments with PARP1 inhibitors.

## Abbreviations:

PAR: – poly(ADP-ribose),
NAD^+^: – nicotinamide adenine dinucleotide,
PARP1: – poly(ADP-ribose) polymerase-1,
AIF: – apoptosis inducing factor,
MOMP: – mitochondrial outer membrane permeabilization,
PAAN: – parthanatos-associated apoptosis-inducing factor nuclease,
MIF: – macrophage migration inhibitor factor,
MPTP: – 1-methyl-4-phenyl-1,2,3,6-tetrahydropyridine,
AIMP2: – aminoacyl-tRNA synthetase complex interacting multi-functional protein-2,
MCAO: – middle cerebral artery occlusion,
AD: – Alzheimer's disease,
HD: – Huntington's disease,
ALS: – amyotrophic lateral sclerosis,
NAMPT: – nicotinamide phosphoribosyltransferase,
MMP: – mitochondrial membrane permeabilization,
MIMP: – mitochondrial inner membrane permeabilization,
DBC1: – protein deleted in breast cancer 1.
